# Evaluation of white sweet potato tube-feeding formula in elderly diabetic patients: a randomized controlled trial

**DOI:** 10.1186/s12986-019-0398-8

**Published:** 2019-10-16

**Authors:** Chiao-Ming Chen, Chun-Kuang Shih, Yi-Jing Su, Kuan-Un Cheang, Shu-Fang Lo, Sing-Chung Li

**Affiliations:** 1 Department of Food Science, Nutrition, and Nutraceutical Biotechnology, Shih-Chien University, No.70, Dazhi St., Zhongshan Dist., Taipei City, 10462 Taiwan; 2 School of Nutrition and Health Sciences, College of Nutrition, Taipei Medical University, 250 Wu-Hsing Street, Taipei, 11031 Taiwan; 3 Department of Agronomy, Chiayi Agricultural Experiment Station, Taiwan Agricultural Research Institute, 2 Min-Cheng Road, Chiayi, 60044 Taiwan

**Keywords:** White sweet potato, Type 2 diabetes mellitus, Tube feeding, Enteral nutrition

## Abstract

**Background:**

Elderly people with type 2 diabetes mellitus (T2DM) have an increased risk of diabetes-related microvascular and macrovascular complications, thus diabetic patients with a functioning gastrointestinal tract but without sufficient oral intake require enteral nutrition (EN) formulas to control blood glucose. White sweet potato (WSP) was a kind of sweet potato could provide a healthy carbohydrate source to EN formula. The aim of this study was to examine at risk of malnutrition T2DM patients whether a WSP-EN would attenuate glucose response and elevate nutritional index compared to a standard polymeric formulas.

**Methods:**

In this randomized, parallel, placebo-controlled, pilot clinical trial to investigate the effects of EN with WSP on aged residents with T2DM in long-term care institutions. In total, 54 eligible participants were randomly assigned to either the non-WSP-EN or WSP-EN group. For 60 days, the WSP-EN group received a WSP formula through nasogastric tube via a stoma with a large-bore syringe. The participants received EN of standard polymeric formulas without WSP in the non-WSP-EN group.

**Results:**

The body weight, body mass index, Mini Nutritional Assessment score, and Geriatric Nutritional Risk Index were significantly higher in the WSP-EN group (*p* < 0.05). Moreover, the WSP-EN intervention reduced glycated hemoglobin levels (6.73% ± 1.47% vs. 6.40% ± 1.16%), but increased transferrin (223.06 ± 38.85 vs. 245.85 ± 46.08 mg/dL), high-density lipoprotein cholesterol (42.13 ± 10.56 vs. 44.25 ± 8.43 mg/dL), and vitamin A (2.45 ± 0.77 vs 2.74 ± 0.93 μM) levels (*p* < 0.05). In addition, there was no important side effects including gastrointestinal intolerance with prescribed doses in our WSP-EN treated patients when compared with control ones.

**Conclusions:**

The results suggest WSP incorporated into enteral formulas can improve nutrition status and glycemic control in elderly diabetic patients.

**Trial registration:**

NCT02711839, registered 27 May 2015.

## Introduction

According to a report of International Diabetes Federation (IDF) in 2015, the prevalence of type 2 diabetes mellitus (T2DM) in the elderly was around 15% on average and exceeded 20% in developed countries like USA and Taiwan [1]. Elderly people with T2DM have an increased risk of traditional diabetes-related microvascular and macrovascular complications, such as retinopathy, nephropathy, neuropathy, stroke, and heart disease [2, 3]. Vascular complications often occur in elderly patients; additionally, T2DM-related geriatric syndromes, such as cognitive impairment, depression, urinary infection, falling, polypharmacy, and sarcopenia, tend to accompany aging [4]. Some diabetic patients with a functioning gastrointestinal tract but without sufficient oral intake require tube feeding (TF) for nutritional support for controlling blood glucose concentrations [5]. A systematic review demonstrated that suitable diabetes-specific formulas (DSFs) as enteral nutrition support through oral supplements or tube feeding are associated with improved glycemic control in patients with diabetes [6, 7].

Sweet potatoes contain considerable quantities of nutrients, including carbohydrates, carotenoids, dietary fiber, anti-oxidants, vitamins and minerals; the tuber contain no saturated fat or cholesterol [8]. Compared with potatoes, sweet potatoes have a lower glycemic index; in other words, they tend to increase blood sugar levels at a relatively slow pace [9]. They also contain fewer calories and complex carbohydrates but more vitamin A and fiber than do potatoes. White sweet potatoes (WSPs; *Ipomoea batatas* L.) of the Convolvulaceae family are used as a healthy source of carbohydrate and are part of traditional medicine in Brazil, Japan, Indonesia and Taiwan [10, 11]. In patients with T2DM, the tuberous root of WSP effectively reduces insulin resistance and fibrinogen, fasting plasma glucose, and low-density lipoprotein cholesterol (LDL-C) levels, but increases adiponectin levels [12–14]. In a recent study by our team, the WSP meal replacement demonstrated a 5% decrease in body weight, body fat, body mass index, and mid-arm circumference and a 3.5% decrease in glycated hemoglobin levels in overweight subjects [15].

Enteral nutrition (EN) formulas are prescribed to elderly patients, when it is necessary, as an exclusive diet or in combination with other foods to achieve recommended dietary intakes. EN formulas are considered medical foods by the U.S. Food and Drug Administration (FDA) and are specially formulated and processed for a patient who requires specific dietary management [16]. Regarding EN, enteral TF refers to the delivery of nutritionally complete feed nutrients directly into the gut through a tube into the stomach, duodenum, or jejunum via the nose, mouth, or direct percutaneous route [17]. The standard polymeric formulas for patients receiving EN include appropriate proportions of carbohydrates, proteins, fats, vitamins, and minerals to support the patients’ medical therapy. Thus far, the main energy substrates used in enteral standard formulas have been maltodextrins, which ensure the flowrate needed for fine-bore TF. Corn maltodextrin and corn syrup solids are the common carbohydrate sources used in enteral formulas [16, 18]. However, although glucose from digested maltodextrins is rapidly absorbed in the small intestine, the increased use of maltodextrins has raised questions regarding its hyperglycemic effects on metabolism and health [19].

Research exploring tuber as an ingredient in EN formula selection to meet specific requirements in diabetic patients has been scant. In addition, most of the contrast between DSFs and standard nutritional supplements used different macronutrients composition contributing to total energy content [20]. Evidence specifically supporting the use of WSP-EN for glycemic control, particularly in elderly patients with T2DM, has been notably limited. The aim of this study was to examine at risk of malnutrition T2DM patients whether a carbohydrate source from WSP would attenuate glucose response and elevate nutritional index compared to a standard polymeric formulas with similar energy content and similar macronutrients contribution. Therefore, our pilot study assessed the potential application of WSP-EN on glucose regulation and anthropometric characteristics in elderly residents with T2DM in long-term care institutions.

## Methods

### Subjects

In total, 100 residents were recruited from long-term care institutions in Keelung County, Taipei City, and Taoyuan County, all in Northern Taiwan, from June 2015 to June 2017. All participants were diagnosed as having T2DM by their physician, regularly taking antidiabetic drugs, and aged 50–80 years. Any participant using oral or injected steroids, or abusing hypnotics or other such drugs was excluded. Moreover, those with acute illnesses, such as recent myocardial infarction, upper or lower gastrointestinal tract bleeding, or poor control of blood glucose (glycated hemoglobin [HbA1c] > 8.5%), and heart failure, were excluded. We also excluded those who could not tolerate EN; were taking nutritional supplements; or had malignancy, endocrine disease (other than T2DM), congestive heart failure, liver cirrhosis, renal insufficiency (serum creatinine (Cr) > 3.0 mg/dL), or moderate anemias (hemoglobin < 9 g/dL), psychosis, or depression. All participants provided informed written consent, along with basic anthropometric measurements: body weight (BW), body height, body mass index (BMI), mid-arm circumference (MAC), mid-arm muscle circumference (MAMC), calf circumference (CC), triceps skinfold (TSF), blood pressure, and pulse rate. Before participation, the participants or legal representatives were offered the opportunity to discuss any queries with the primary investigator, the physician, and the study coordinator. Taipei Medical University approved the study protocol (TMU-JIRB 201505027); this trial is registered at ClinicalTrials.gov (NCT02711839).

### Trial design

**Fig. 1:**
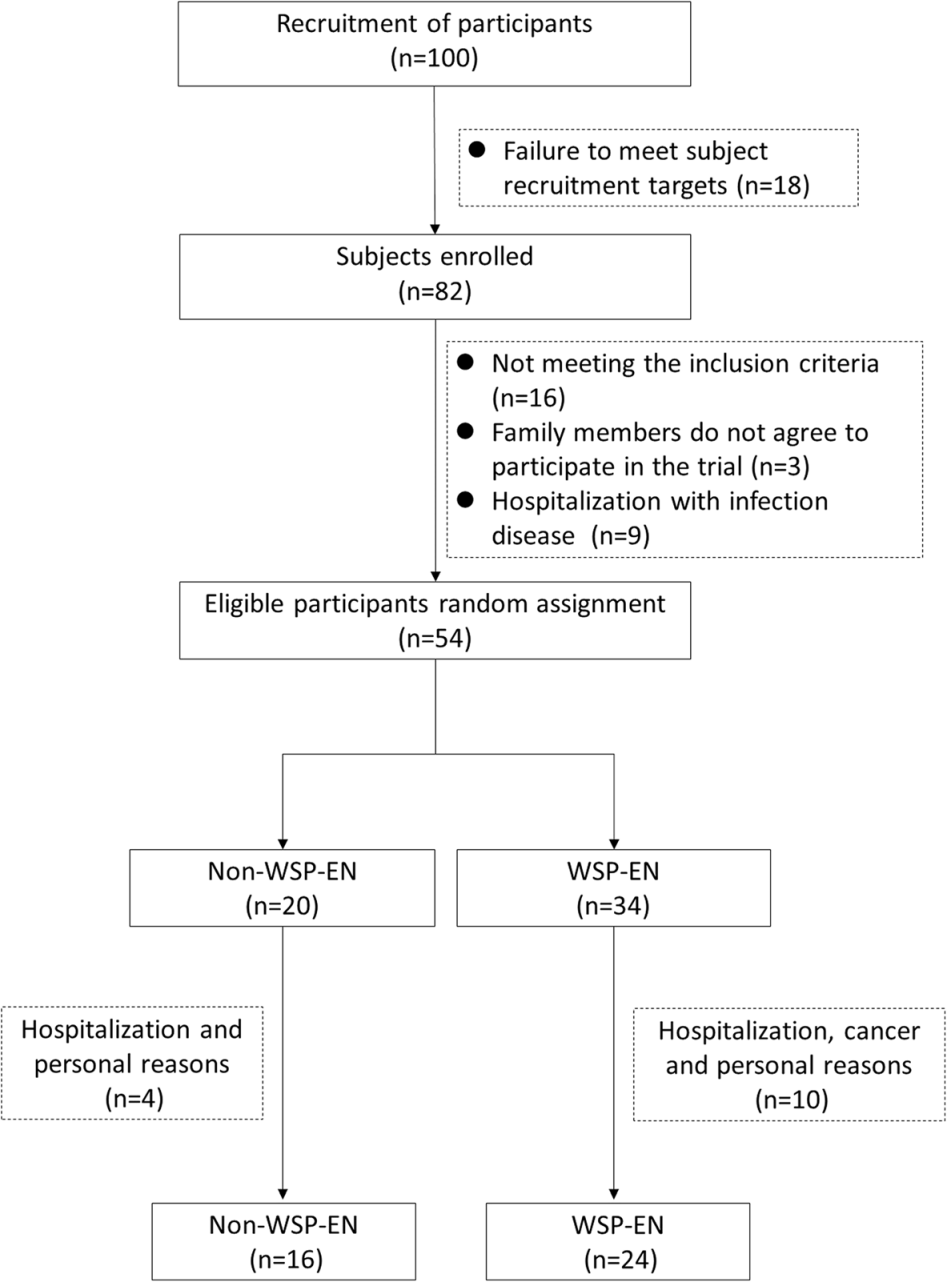
Flowchart of participant selection

This was a randomized, parallel, placebo-controlled, pilot clinical trial. One hundred subjects in this clinical trial recruitment showed in Fig. 1. Of the enrolled 82 individuals, 16 were excluded for not meeting the inclusion criteria, 3 because of family disagreements, and 9 for being hospitalized for infections. Next, the remaining 54 participants were randomly allocated to either the non-WSP-EN (control) or WSP-EN (treatment) group. Four individuals from the non-WSP-EN group withdrew from the study because they were hospitalized or had poor compliance. Another ten individuals were excluded, because during follow-up, they were hospitalized, had cancer, or had poor compliance in the WSP-EN group. Study meals were tailored to meet daily energy need of each subject, calculated using the information of anthropometry and daily physical activity level by a registered dietitian. All the formulas were added to warm water to provide a calorie density of 1 kcal/mL. Each participant was administered 5–6 times per day by bolus nasogastric feeding. The WSP-EN group received an average five packets of WSP formula (each packet had 20 g of WSP in 70 g of formula). All participants did not consume other foods except for tube feeding in long-term care institutions.

During the 60-day clinical trials, in order to avoid abnormal blood glucose caused by the test diet, all subjects took blood samples daily from the fingertips within 3 days after WSP-EN intervention and once a week to ensure that the subjects did not occur acute hyperglycemia or hypoglycemia. We also recorded the modification of drug or insulin dosage due to abnormal blood glucose during the experiment period. The gastrointestinal tract tolerance of subjects, including diarrhea, constipation, vomiting, bloating, and gastric residue were recorded during the trial.

### Study diets

The special sweet potato cultivar WSP [Tainung 10 (TNG 10)] was cultivated by an agricultural institution in Taiwan. WSP-EN contains 28.6% (by weight) of TNG10 and adds whey proteins, vegetable fat, compound vitamins, and minerals to make it a standard polymeric formulas comparable to non-WSP-EN. A registered clinical dietitian validated the total energy, macro and micro nutrients meeting the American Diabetes Association’s guideline [21]. The percentages of calories from carbohydrates, proteins, and fats in the WSP formula were 53.4, 19.5, and 27.1%, respectively. Each 100 g of the WSP-EN formula contains 428.3 kcal, 20.9 g of protein (AOAC 990.03 method), 12.9 g of fat (CNS5036 method), 60.3 g of carbohydrate, 13.5 g of sugar (CNS12634 method) and 6.3 g of dietary fiber (AOAC 985.29 method). This ingredient analysis was validated by Food Industry Research and Development Institute, Taiwan. The product of WSP-EN was manufactured by Huei Jian Sucare Ltd., Taiwan. In the non-WSP-EN group, the participants received nasogastric EN by institutional feeding for standard polymeric formulas, with similar total calorie content and appropriate proportion of macronutrients composition (Table 1).

**Table 1:** Nutrient composition of study formulas per 100 g

Nutrient	Non-WSP-EN	WSP-EN
Calories (kcal)	443.3	428.3
Carbohydrates (g) (% of energy)	59.7 (52.1)	60.3 (53.4)
Fiber (g)	4.0	6.3
Sugars (g)	2.6	13.5
Protein (g) (% of energy)	17.8 (16.1)	20.9 (19.5)
Fat (g) (% of energy)	15.7 (31.9)	12.9 (27.1)
MUFA (g)	7.6	9.1
PUFA (g)	5.1	1.9
SFA (g)	3.3	1.9

Where indicated, percentages of total calories are given in parentheses. Non-WSP-EN was the standard polymeric formula used in various standard polymeric formulas designed to support diabetic patients, calculated from the average value of EN product from individualized used.

*MUFA* monounsaturated fatty acids, *PUFA* polyunsaturated fatty acids, *SFA* saturated fatty acids

### Nutritional risk assessment

We use the Taiwanese version of Mini Nutritional Assessment (MNA) to assess the nutritional status of long-lived elderly people and to investigate the nutritional status of residents in long-term care institutions. The assessment form includes weight changes, mobility, stress disorder, mental state and body mass index. Malnutrition indicator score were, for normal nutritional status (24 ≤ MNA), at risk of malnutrition (17 ≤ MNA ≤ 23.5), and malnourished (< 17) [22]. The Geriatric Nutritional Risk Index (GNRI) is an index for evaluating at-risk elderly medical patients. The GNRI formula is as follows: GNRI = (1.489 × albumin (g/L)) + (41.7 × (current body weight / ideal body weight)). Risk of mortality and risk of complications were, respectively, for major nutrition-related risk (GNRI < 82), for moderate nutrition related risk (82 ≤ GNRI < 92), for a low nutrition-related risk (92 ≤ GNRI ≤98), and for no risk of malnutrition (GNRI > 98) [23].

### Biochemical analyses

Blood samples from all participants were measured after 12 h of fasting at the beginning of and after the 60-day intervention. Blood was obtained through capillary blood collection, and blood sugar was examined instantly with an OneTouch FreeStyle Freedom Lite blood glucose monitor (Abbott Diabetes Car Inc., CA, USA). Other blood biomarkers, such as total cholesterol (TC), triglyceride (TG), high-density lipoprotein cholesterol (HDL-C), low-density lipoprotein cholesterol (LDL-C), aspartate aminotransferase (AST), alanine aminotransferase (ALT), Creatinine (Cr), and uric acid levels were analyzed from a 10-mL blood sample on Beckman Synchron LX-20 (Beckman Coulter Inc., CA, USA). Serum insulin was determined by an electrochemiluminescence immunoassay (Roche, Switzerland), and inter-day coefficient of variation (CV) was 8%. Homeostasis model assessment-Insulin Resistance (HOMA-IR) was calculated according to the formula: insulin × glucose/22.5, with insulin expressed as μU/mL and glucose as mmol/L [24] . HbA1c was analyzed from another 1.5-mL blood sample by using Spotchem SP-4410 (Arkray Inc., Kyoto, Japan). High sensitivity C-reactive protein (Hs-CRP) in serum was determined in TBA-40FR chemistry analyzer using a latex-enhanced turbidimetric immunoassay (Toshiba, Japan), and inter-day CV was 7.2%. Plasma retinol and α-tocopherol was determined using a reverse-phase HPLC method, according to Bieri et al. [25]. Briefly, two antioxidant vitamins in plasma were extracted with hexane and then quantified using an HPLC system (Hitachi, Japan) equipped with a LiChroCART C-18 column (4 × 250 nm, 4 μm; Perkin-Elmer, West Lafayette, IN) and a UV/VIS detector (Hitachi, Japan). The concentration in unknown samples was calculated using a standard curve constructed using authentic retinol and α-tocopherol.Intra-day and inter-day CV was 7.5 and 11.3%, respectively.

### Statistical analyses

The sample size was calculated using the HbA1c data of our previous study [26]. A sample size of 54 subjects was needed to detect the significant difference in HbA1c at the α value of 0.05 and power 0.95 by G power 3.1. Statistical analyses were performed using SPSS19 (version 19; SPSS Inc., Chicago, IL, USA). For intragroup testing, we performed the paired *t* test with the Wilcoxon signed-rank test to compare the differences in participants’ dietary treatment, anthropometric parameters, glycemic markers, lipid profiles, and nutrition indexes in each group. For intergroup comparisons, we performed the independent *t* test with the Mann–Whitney *U* test to compare the final values or nets between the non-WSP-EN and WSP-EN groups. All data are expressed as means ± standard deviations (SDs). The results were considered statistically significant for *p* < 0.05.

## Results

### Characteristics of participants

We initially recruited 100 participants; however, after professional evaluation by physicians and clinical dietitians, only 54, who were eligible, completed in the study. Fourteen participants dropped out: four non-WSP-EN participants due to hospitalization, personal reasons and ten WSP-EN participants because of infection, cancer and personal reasons (Fig. 1). The overall response rate was 74% (80 and 71% in the non-WSP-EN and WSP-EN groups, respectively). In the WSP-EN and non-WSP-EN groups, the average age was 66.7 ± 13.4 and 68.4 ± 12.8 years, respectively, and the average diabetes duration was 6.6 ± 3.9 and 9.6 ± 6.5 years, respectively. Basic patient characteristics such as sex, age, height, diabetes duration, antidiabetic, antihypertensive, and lipid-lowering drugs did not differ significantly (Table 2).

**Table 2:** Baseline participant characteristics

Characteristic	Non-WSP-EN	WSP-EN
Male/female (n)	8/8	13/11
Age (year-old)	68.4 ± 12.8	66.7 ± 13.4
Height (cm)	154.3 ± 8.8	154.9 ± 9.6
Duration of diabetes (years)	9.6 ± 6.5	6.6 ± 3.9
Medication		
Anti-diabetic drugs (%, n)	68.8% (11)	79.2% (19)
Insulin (n)	1	8
Sulfonylureas (n)	1	4
Meglitinide (n)	2	3
Biguanide (n)	7	11
α-glucosidase inhibitors (n)	2	1
Thiazolidinedione (n)	0	0
DPP-4 inhibitors (n)	2	1
Anti-hypertensive drugs (%, n)	56.3% (9)	62.5% (15)
Lipid lowering drugs (%, n)	12.5% (2)	8.3% (2)

All data are expressed as means ± standard deviations or percentage. Analyzed using the independent-sample *t* test or chi-square test. There was no significant different in two group (*p* > 0.05) DPP-4, dipeptidyl peptidase-4

### Dietary treatment and compliance

Since both WSP-EN and non-WSP-EN have similar calories and macronutrients composition (Table 1), the calorie intake before (Day 0) and after (Day 60) intervention did not differ significantly between the two groups in Table 3. Non-WSP-EN significantly increased carbohydrate intake (194.39 ± 23.72 vs. 201.21 ± 22.45 g), but reduced fat intake (54.74 ± 4.38 vs. 50.43 ± 4.96 g); by contrast, WSP-EN significantly increased protein intake (58.01 ± 7.96 vs. 70.57 ± 7.14 g) and dietary fiber intake (15.70 ± 6.28 vs. 21.43 ± 2.24 g), but reduced fat intake (55.07 ± 7.01 vs. 43.88 ± 4.58 g). Moreover, changes in fat uptake were significantly lower in the WSP-EN group than in the non-WSP-EN group (− 4.31 ± 4.96 vs. − 11.19 ± 5.40 g), whereas that in protein uptake (2.85 ± 5.49 vs 15.50 ± 15.30 g) and dietary fiber uptake (− 0.55 ± 3.10 vs 5.73 ± 6.46 g) was significantly higher in the WSP-EN group than in the non-WSP-EN group. No significant differences were noted in compliance between the two groups. Both EN formulas were gastrointestinal tolerance; however, two non-WSP-EN and four WSP-EN group participants reported flatulence and constipation in the first week of intervention. No other adverse event was reported in both groups.

**Table 3:** Changes in calories, macronutrients and dietary fiber intake from day 0 to day 60 in elderly T2DM subjects

	Non-WSP-EN			WSP-EN		
	Day 0	Day 60	Changes	Day 0	Day 60	Changes
Calories (kcal)	1487.50 ± 144.34	1493.75 ± 142.45	6.25 ± 25.00	1525.00 ± 174.46	1500.00 ± 156.73	− 25.00 ± 69.16
Carbohydrate (g)	194.39 ± 23.72	201.21 ± 22.45 *	6.82 ± 8.75	205.19 ± 27.56	205.10 ± 21.43	− 0.09 ± 15.77
Protein (g)	58.97 ± 8.46	61.82 ± 8.32	2.85 ± 5.49	58.01 ± 7.96	70.57 ± 7.14 *	15.50 ± 15.30 ^#^
Fat (g)	54.74 ± 4.38	50.43 ± 4.96 *	−4.31 ± 4.96	55.07 ± 7.01	43.88 ± 4.58 *	−11.19 ± 5.40 ^#^
Dietary fiber (g)	12.44 ± 4.40	11.89 ± 3.27	− 0.55 ± 3.10	15.70 ± 6.28	21.43 ± 2.24 *	5.73 ± 6.46 ^#^

Data are presented as means ± standard deviations.

* Significant within-group difference after intervention (paired t test analysis; *p* < 0.05). # Significant between-groups difference after intervention (independent t test; *p* < 0.05)

### Anthropometric characteristics, nutrition indexes and clinical biochemistry

In the non-WSP-EN group, BW, BMI, CC, MAC, MAMC, TSF, MNA score, and GNRI did not significantly differ before and after intervention. By contrast, after the 60-day intervention, WSP-EN had increased MNA score (18.85 ± 3.71 vs. 20.13 ± 3.59), and GNRI score (46.25 ± 7.95 vs. 47.40 ± 8.14) in Table 4. Regarding post-intervention intergroup differences, although the differences in the MNA scores were not significantly difference, the GNRI was significantly higher in the WSP-EN group than in the non-WSP-EN group (− 0.01 ± 1.88 vs. 1.15 ± 1.27), implying that higher nutrients support provided by WSP-EN formula.

**Table 4:** Changes in anthropometric parameters and nutrition indexes from day 0 to day 60 in elderly T2DM subjects

	non-WSP-EN			WSP-EN		
	Day 0	Day 60	Changes	Day 0	Day 60	Changes
BW (kg)	54.89 ± 7.40	54.88 ± 7.19	− 0.02 ± 2.27	51.56 ± 9.29	52.83 ± 9.28 *	1.27 ± 1.40
BMI (kg/m^2^)	23.14 ± 3.39	23.15 ± 3.46	0.01 ± 0.94	21.62 ± 4.13	22.17 ± 4.27 *	0.55 ± 0.62
CC (cm)	26.70 ± 4.07	26.88 ± 4.30	0.18 ± 1.76	26.18 ± 4.01	25.94 ± 3.85	− 0.24 ± 1.19
MAC (cm)	26.42 ± 2.33	26.91 ± 2.99	0.49 ± 3.34	25.51 ± 3.72	25.63 ± 3.23	0.12 ± 1.93
MAMC (cm)	21.64 ± 3.44	21.71 ± 2.34	0.08 ± 3.61	21.12 ± 3.09	21.35 ± 2.51	0.23 ± 2.26
TSF (mm)	15.25 ± 6.80	16.51 ± 5.97	1.26 ± 3.52	13.98 ± 6.87	13.61 ± 5.00	−0.37 ± 4.24
MNA (score)	20.78 ± 1.67	20.69 ± 2.85	−0.09 ± 2.77	18.85 ± 3.71	20.13 ± 3.59 *	1.27 ± 1.43
GNRI (score)	49.17 ± 6.51	49.16 ± 6.69	−0.01 ± 1.88	46.25 ± 7.95	47.40 ± 8.14 *	1.15 ± 1.27 ^#^

Data are presented as means ± standard deviations.

*Significant within-group difference after intervention (paired t test analysis; *p* < 0.05).

#Significant between-groups difference after intervention (independent t test; *p* < 0.05).

*BMI* body mass index; *CC* calf circumference; *MAC* mid-arm circumference; *MAMC* mid-arm muscle circumference; *TSF* triceps skinfold; *MNA* Mini Nutritional Assessment; *GNRI* geriatric nutritional risk index.

*p < 0.05 pre- vs. post-treatment, paired *t* test.

^#^*p* < 0.05 between non-WSP-EN vs. WSP-EN group, independent-sample *t* test

### Glycemic status and lipid profile

At baseline, both groups demonstrated good glycemic control due to antidiabetic drugs. After the 60-day intervention, the WSP-EN group had stable fasting blood glucose levels (119.08 ± 61.23 vs. 122.63 ± 57.94 mg/dL); however, the non-WSP-EN group demonstrated a significant increase in fasting blood glucose levels (108.19 ± 28.05 vs. 139.13 ± 51.07 mg/dL). Nevertheless, WSP-EN for 60 days reduced HbA1c levels (6.73% ± 1.47% vs. 6.40% ± 1.16%). No significant intergroup and intragroup differences were noted for the glycemic markers, namely insulin and HOMA-IR. Intergroup and intragroup analyses of lipid profiles indicated no statistical differences in TC, triglycerides, and LDL-C levels and in the TC/HDL-C and LDL-C/HDL-C ratios. Interestingly, WSP-EN increased HDL-C levels by 5.1% (42.13 ± 10.56 vs. 44.25 ± 8.43 mg/dL) (Table 5).

**Table 5:** Changes in glycemic markers and lipid profiles from day 0 to day 60 in elderly T2DM subjects

	Non-WSP-EN			WSP-EN		
	Day 0	Day 60	Changes	Day 0	Day 60	Changes
FBG (mg/dL)	108.19 ± 28.05	139.13 ± 51.07 *	30.94 ± 38.50	119.08 ± 61.23	122.63 ± 57.94	3.54 ± 59.49
HbA1c (%)	5.84 ± 0.59	6.05 ± 0.97	0.21 ± 0.60	6.73 ± 1.47	6.40 ± 1.16 *	−0.33 ± 0.75 ^#^
Insulin (μU/mL)	7.88 ± 6.78	10.98 ± 6.33	3.11 ± 7.06	8.72 ± 6.63	18.47 ± 13.83	9.75 ± 3.14
HOMA-IR	2.26 ± 2.77	3.41 ± 2.39	1.15 ± 2.82	2.92 ± 3.54	6.94 ± 4.62	4.01 ± 4.49
TC (mg/dL)	148.94 ± 25.42	158.38 ± 33.15	9.44 ± 26.65	159.29 ± 25.69	163.67 ± 26.96	4.38 ± 17.32
TG (mg/dL)	157.63 ± 82.40	161.44 ± 79.30	3.81 ± 57.85	127.21 ± 54.99	140.04 ± 72.52	12.83 ± 41.88
HDL-C (mg/dL)	38.19 ± 12.39	37.88 ± 8.15	− 0.31 ± 6.80	42.13 ± 10.56	44.25 ± 8.43 *	2.13 ± 4.93
LDL-C (mg/dL)	91.63 ± 18.11	99.69 ± 25.82	8.06 ± 20.97	100.96 ± 23.37	106.71 ± 23.14	5.75 ± 14.89
TC/ HDL-C	4.18 ± 1.12	4.39 ± 1.38	0.21 ± 0.71	3.99 ± 1.09	3.82 ± 0.84	−0.17 ± 0.61
LDL-C/HDL-C	2.62 ± 0.85	2.80 ± 1.07	0.17 ± 0.64	2.56 ± 0.91	2.49 ± 0.66	−0.07 ± 0.52

Data are presented as means ± standard deviations.

* Significant within-group difference after intervention (paired t test analysis; *p* < 0.05).

# Significant between-groups difference after intervention (independent t test; *p* < 0.05).

*FBG* Fasting blood glucose; *HbA1c* glycated hemoglobin; *HOMA-IR* homeostatic model assessment–insulin resistance. *TC* Total cholesterol; *TG* triglyceride; *HDL-C* high-density lipoprotein cholesterol; *LDL-C* low-density lipoprotein cholesterol.

*p < 0.05 pre- vs. post-treatment, paired *t* test.

^#^*p* < 0.05 between non-WSP-EN vs. WSP-EN group, independent-sample *t* test

### Blood biomarkers and nutritional indexes

The non-WSP-EN group demonstrated significant increase in the concentrations of Hs-CRP (0.78 ± 0.97 vs. 1.68 ± 1.95 mg/dL), but the WSP-EN group did not. Between the non-WSP-EN and WSP-EN groups, biochemical metabolic index markers, such as albumin, Cr, and BUN demonstrated no significant differences. Prealbumin changes was significantly higher in the WSP-EN group than in the non-WSP-EN group (− 0.89 ± 3.03 vs. 1.06 ± 2.81 mg/dL). Uric acid concentration was significantly lower in WSP-EN group versus non-WSP-EN group (− 0.30 ± 0.71 vs. -0.89 ± 0.92 mg/dL). WSP-EN led to a 10.2% increase in serum transferrin concentration (223.06 ± 38.85 vs. 245.85 ± 46.08 mg/dL) and thus a better nutrition status, reflected by the changes in the serum transferrin concentration (1.54 ± 17.32 vs 22.78 ± 16.74). WSP-TP, but not non-WSP-EN, also increased vitamin A (2.45 ± 0.77 vs 2.74 ± 0.93 μM). The liver function indices such as AST and ALT in WSP-EN are within in normal range (Table 6).

**Table 6:** Changes in blood biomarkers and nutritional indexes from day 0 to day 60 in elderly T2DM subjects

	Non-WSP-EN			WSP-EN		
	Day 0	Day 60	Changes	Day 0	Day 60	Changes
AST (U/L)	25.75 ± 15.41	46.00 ± 86.46	20.25 ± 88.42	26.96 ± 16.96	24.25 ± 9.23	−2.71 ± 10.59
ALT (U/L)	22.19 ± 15.55	33.00 ± 49.40	10.81 ± 50.23	24.08 ± 25.52	21.00 ± 13.21	−3.08 ± 16.69
Pre-Alb (mg/dL)	22.86 ± 4.30	21.96 ± 5.70	−0.89 ± 3.03	23.63 ± 6.30	24.70 ± 6.04	1.06 ± 2.81 ^#^
Albumin (g/dL)	3.56 ± 0.24	3.53 ± 0.25	−0.03 ± 0.17	3.54 ± 0.38	3.61 ± 0.35	0.07 ± 0.23
Cr (mg/dL)	0.62 ± 0.19	0.64 ± 0.22	0.03 ± 0.07	0.76 ± 0.30	0.73 ± 0.27	− 0.03 ± 0.11
BUN (mg/dL)	13.31 ± 5.51	14.75 ± 5.98	1.44 ± 4.24	18.50 ± 11.73	20.58 ± 9.45	2.08 ± 6.70
Uric acid (mg/dL)	5.71 ± 1.61	5.41 ± 1.58	− 0.30 ± 0.71	5.60 ± 1.85	4.71 ± 1.59 *	− 0.89 ± 0.92 ^#^
Transferrin (mg/dL)	237.68 ± 48.98	239.23 ± 51.97	1.54 ± 17.32	223.06 ± 38.85	245.85 ± 46.08 *	22.78 ± 16.74 ^#^
Hs-CRP (mg/dL)	0.78 ± 0.97	1.68 ± 1.95 *	0.90 ± 1.50	0.69 ± 0.59	0.76 ± 0.64	0.07 ± 0.75
Vitamin A (μM)	2.82 ± 0.35	2.71 ± 0.49	−0.11 ± 0.21	2.45 ± 0.77	2.74 ± 0.93 *	0.29 ± 0.46
Vitamin E (μM)	33.06 ± 7.62	34.31 ± 8.93	1.25 ± 2.63	29.04 ± 6.68	31.23 ± 11.33	2.18 ± 8.50

Data are presented as means ± standard deviations.

* Significant within-group difference after intervention (paired t test analysis; *p* < 0.05).

# Significant between-groups difference after intervention (independent t test; *p* < 0.05).

*AST* aspartate aminotransferase; *ALT* alanine aminotransferase; *Pre-Alb* Pre-albumin; *BUN* blood urea nitrogen; *Hs-CRP* high-sensitivity C-reactive protein.

*p < 0.05 pre- vs. post-treatment, paired *t* test.

^#^*p* < 0.05 between non-WSP-EN vs. WSP-EN group, independent-sample *t* test

## Discussion

Elderly patients with T2DM in long-term care institutions who have poor glycemic control may have increased risks of geriatric syndromes, such as stroke, heart disease, retinopathy, kidney disease, skin lesions, depression, cognitive impairment, hyperglycemia coma [27, 28]. However, the administration of a DSF could provide better glycemic control and clinical outcomes for improving metabolic control and clinical outcomes [29]. Though some subjects withdrew from the study for health or personal reasons, resulting in a small sample size at the end of the clinical trial, we still observed a glycaemic improvement effect in the WSP-EN group. During the 60 days of clinical trials, in one participant, insulin injection was discontinued after 15 days of intervention to avoid hypoglycemia.

Due to higher protein and lower fat composition than standard polymeric formulas, the well-developed reduced fat intake and increased protein intake in subjects. The anthropometric parameters such as BW, BMI, MNA and GNRI were significantly higher during WSP-EN intervention. In addition, WSP-EN reduced HbA1c by 4.9% and increased HDL-C by 5.1% after the 60-day intervention. The useful method for analysis subjects’ body composition were Anthropometry, Dual-energyX-ray absorptiometry and Computed tomography [30]. The main determinants of energy expenditure are body size and body composition, food intake and physical activity [31]. Because of our subjects were elderly, chronic bed rest and chronic disabilities in long-term care institutions (not hospitalized objects), for its limitation, we assessed their energy requirement according to the information of anthropometry and daily physical activity level by an experienced registered dietitian.

To our knowledge, this is the first study to report the role of WSP in DSFs for elderly residents with T2DM in long-term care institutions. Although some studies have shown a beneficial effect of high-fat enteral formula on glycemic control [29, 32, 33], others reported no such effect [34, 35]. American Diabetes Association’s nutritional therapy for adults with diabetes recommend a moderate intake of carbohydrate (~ 50% of total calories), protein (15–20% of total calories), fat (~ 30% of total calories), saturated fatty acids < 7% of total energy, and fiber intake (14 g of fiber/1000 kcal) [21]. Nevertheless, the clinical practice guidelines do not use additional recommendations to prevent or control EN patients with hyperglycemia, the rational amount of carbohydrate intake for older people with diabetes has been scant.

Suppression of hyperglycemia by WSP may be explained by the following mechanism. According to Kusano et al., the WSP extract contains a distinct glycoprotein, which can effectively reduce the blood insulin concentration in mice with streptozotocin (STZ)-induced diabetes [36, 37]. Oki et al. indicated that WSP, containing 5% of this distinct glycoprotein, could effectively reduce fasting blood glucose levels, increase glucose and insulin sensitivity, and finally, improve insulin resistance in diabetic rats [38]. The methanol extract of WSP can also reduce the blood glucose levels, attenuating oxidative stress and dyslipidemia in rats with STZ-induced diabetes [11]. Royhan et al. demonstrated that WSP produces hypoglycemic activity by inducing pancreatic beta cell regeneration and increasing insulin expression in diabetic rats [39]. In clinical patients with T2DM, high doses of WSP (4 g) before breakfast, lunch, and dinner for 6 weeks reduced fasting plasma glucose, total cholesterol, and LDL-C levels [14]. Our previously study also showed that WSP meal replacements can facilitate individual weight loss and lower fasting blood glucose in overweight workers [15]. Ozaki et al. showed that white sweet potato is a (1→3)-β-D-galactan highly branched at O-6 with (1→6)-β-D-galactan, in which the branched chains are substituted at the O-3 position with α-L-Araf-(1→and α-L-Araf-(1→5)-α-L-Araf-(1→and at the O-6 position typically with α-L-Rhap-(1→4)-β-D-GlcAp-(1→as terminating groups [10], resulting in significantly decreased plasma glucose in spontaneous diabetic mice [38].

The dental problems, dysphagia and change in their taste may also act negatively such as malnourished on elderly. When patients have to use a replacement EN formula to nutrition therapy, the clinical symptoms often appear in the form of gastrointestinal symptoms, such as abdominal distension and diarrhea [40]. In patients with T2DM, these symptoms may arise in any region of the alimentary tract; nevertheless, the common symptoms are heartburn, nausea, vomiting, diarrhea, constipation, fecal incontinence, and abdominal pain [41]. Diarrhea, also a major concern during EN, may represent a limitation to the broad use this therapy [40]. The WSP-EN group did not show any abnormal gastrointestinal symptoms after 60-day treatment in our study. At the beginning of our current experiment, three participants experienced bloating because the fiber content in the WSP formula was significantly higher than that in the previously used EN formula; this problem was alleviated after 2 weeks of continuous feeding. In two female participants, constipation was alleviated after water intake was increased. Two participants had watery and loose stools at the beginning of the study; after WSP-EN intervention, the bowel consistency improved and the feces became solid. Results of a meta-analysis also indicate a lower incidence of diarrhea with use of fiber-containing enteral formula [42].

The anthropometric parameters and nutrition indexes significantly improved may be related to WSP-EN containing more protein. In general, older people appear to require 1.0–1.3 g/kg/day of dietary protein to optimize physical function [43]. At the end of the trial, the WSP-EN group demonstrated an increase in protein intake compared with the non-WSP-EN group (1.3 vs. 1.1 g/kg/day). A EN formula or diet containing high-protein and low-carbohydrate loads can significantly improve glucose control in subjects with type 2 diabetes [44, 45]. The better glycemic control and improved nutrition status were not only WSP but also increased daily protein intake. In addition, the fiber intake in the WSP-EN group was increased by 9.5 g per day than that in the non-WSP-EN group, it also contributed to a decreased HbA1c and a stabilized blood sugar.

We noted another interesting result that was WSP-EN contains more sugar (13.5 g/100 g). That is natural sugar in WSP, not extra added. Although WSP-EN contains more sugar, it does not mean that it will affect blood sugar. In our preclinical trial, we measure plasma glucose response in 20 healthy participants, the average glycemic index was 32.3 for the WSP-EN formula, implying that WSP-EN may contain more fibers to interfere intestinal sugar absorption that give more advantages in terms of glycemic control.

In a previous study of 100 participants with diabetes, β-glucuronidase activity, which indicates the regulation of a noninsulin-sensitive pathway for glucose metabolism such as the glucuronic acid cycle, was significantly higher in participants with diabetes than in the controls group [46]. Wu et al. suggested that when healthy volunteers consumed konjac fiber supplement (4·to 5 g/day) for 4 weeks, that dietary supplement significantly reduced fecal β-glucuronidase activity and fecal secondary bile acid level [47]. Lestari et al. proposed prebiotic components such as fructooligosaccharide (FOS), inulin, raffinose, and others are naturally found in several plants such as sweet potato tubers [48]. The WSP ingredient as a prebiotic may affect the host by selectively stimulating the growth and/or β-glucuronidase activity of one or a limited number of bacteria in the colon. In the present study, the WSP-EN could provide 21.43 g fiber/day with a better glycemic control; thus, dietary fiber has a potential role in diabetes etiology. However, to confirm these results, analyzing the β-glucuronidase activity in stools of our participants are essential.

Additionally, resistant starch (RS) is a promising dietary fiber for the prevention or treatment of colon cancer, diabetes, obesity and its related diseases [49, 50]. Consumption of a meal high in resistant starch or soluble fiber decreases peak insulin and glucose concentrations and areas under the curve (AUCs) [51]. The 20 insulin resistant subjects consumed the RS supplement for 12 weeks that improves insulin sensitivity in metabolic syndrome [52]. In our previous WSP digestibility test, the RS and slowly digestible starch (SDS) contents were higher during WSP food processing [15]. Therefore, whether the higher SDS content in WSP-EN contributes to HbA1c loss in T2DM subjects warrants further research.

The strengths of this study are that it represents WSP as ingredient applied to elderly diabetic patients through nasogastric TF for clinical EN support. The first limitations are the small experimental sample size and short trial duration; both of these may have impeded the detection of significant changes in the study measure. It is difficult to find two group subjects who were completely match in basic characteristics. But, we used the independent-sample t test or chi-square test, the duration of diabetes (years) and anti-diabetic drugs use were not statistically significant (*p* > 0.05). Second, the antidiabetic active ingredients in WSP warrant further clarification through glycoprotein or polysaccharide analysis. Third, we did not measure the intestinal microbiota changes in this study but we conducting other study to evaluate the effect of WSP-EN on intestinal microbium separately.

## Conclusions

The study findings indicate that the consumption of functional carbohydrate from WSP as an ingredient could ameliorate glycemia and improve anthropometric parameters and nutrition indexes in elderly diabetic patients. Although the present study was not conducted in critically ill patients, the results may suggest a potential role of WSP-EN in T2DM population. Further research in a larger sample of patients with diabetes or impaired glucose tolerance is required.

## Data Availability

All information data used in our research are not openly available due to confidentiality of information warranted by the written informed consent.
